# Effects of social approval bias on self-reported fruit and vegetable consumption: a randomized controlled trial

**DOI:** 10.1186/1475-2891-7-18

**Published:** 2008-06-27

**Authors:** Tracy M Miller, Madiha F Abdel-Maksoud, Lori A Crane, Al C Marcus, Tim E Byers

**Affiliations:** 1 Department of Preventive Medicine and Biometrics, University of Colorado Denver, 4200 East 9th Avenue, Denver, CO, 80262, USA; 2 AMC Cancer Research Center, 1600 Pierce St., Denver, CO, 80214, USA

## Abstract

**Background:**

Self-reports of dietary intake in the context of nutrition intervention research can be biased by the tendency of respondents to answer consistent with expected norms (social approval bias). The objective of this study was to assess the potential influence of social approval bias on self-reports of fruit and vegetable intake obtained using both food frequency questionnaire (FFQ) and 24-hour recall methods.

**Methods:**

A randomized blinded trial compared reported fruit and vegetable intake among subjects exposed to a potentially biasing prompt to that from control subjects. Subjects included 163 women residing in Colorado between 35 and 65 years of age who were randomly selected and recruited by telephone to complete what they were told would be a future telephone survey about health. Randomly half of the subjects then received a letter prior to the interview describing this as a study of fruit and vegetable intake. The letter included a brief statement of the benefits of fruits and vegetables, a 5-A-Day sticker, and a 5-a-Day refrigerator magnet. The remainder received the same letter, but describing the study purpose only as a more general nutrition survey, with neither the fruit and vegetable message nor the 5-A-Day materials. Subjects were then interviewed on the telephone within 10 days following the letters using an eight-item FFQ and a limited 24-hour recall to estimate fruit and vegetable intake. All interviewers were blinded to the treatment condition.

**Results:**

By the FFQ method, subjects who viewed the potentially biasing prompts reported consuming more fruits and vegetables than did control subjects (5.2 vs. 3.7 servings per day, p < 0.001). By the 24-hour recall method, 61% of the intervention group but only 32% of the control reported eating fruits and vegetables on 3 or more occasions the prior day (p = 0.002). These associations were independent of age, race/ethnicity, education level, self-perceived health status, and time since last medical check-up.

**Conclusion:**

Self-reports of fruit and vegetable intake using either a food frequency questionnaire or a limited 24-hour recall are both susceptible to substantial social approval bias. Valid assessments of intervention effects in nutritional intervention trials may require objective measures of dietary change.

## Background

Dietary assessment methods based on self-reports are widely used in nutritional intervention studies. Conclusions drawn from these studies may be impeded, however, by the presence of social desirability (the tendency to respond in such a way as to avoid criticism) and social approval (the tendency to seek praise) biases, which are tendencies for an individual to respond in a manner consistent with expected norms [1-3].

Previous dietary studies suggest that survey respondents who exhibit social desirability characteristics are more likely to underreport energy and fat intake [2-6]. Only limited work has been done to determine whether an intervention or knowledge of the goals of an intervention can produce biases using dietary assessment tools common in nutritional epidemiology. Kristal et al, using a brief food frequency measure, elicited significantly lower reports of fat intake from undergraduate students who had been exposed to a brief nutritional message about fats and health than from students not exposed to that message [6].

Cognitive psychologists term the different types of memory required for dietary recalls vs. food frequency questionnaires (FFQs) as specific vs. generic. Specific memory relies on particular memories about episodes of eating and drinking, as in a 24-hour recall. Generic memory relies on general impressions about one's typical diet, as in a FFQ that directs a respondent to report impressions about usual frequency of eating a food over the previous year. As the time between the behavior and the report increases, respondents rely more on generic memory and less on specific memory General memory may be more subject to social approval bias than are specific recalls of actual recent events [7].

Several studies suggest that women's responses to dietary intake questions may be more affected by social desirability bias than those of men [2,3,6]. Hebert et al observed a large underestimate of fat and energy intake associated with increased social desirability score in a 7-day diet recall (cognitively similar to the food frequency questionnaire), when compared with a 24-hour diet recall. For total energy, this bias was approximately twice as large for women as for men [2]. In a later study, Hebert et al found that energy and fat intake were overestimated by men but underestimated by women who had higher levels of social desirability [3]. Kristal et al reported a similar finding in his study of undergraduate students: underreporting of fat intake from a food frequency questionnaire by women, but not by men [6].

In this study, we sought to estimate the magnitude and direction of social approval bias in reported fruit and vegetable intake obtained from a short food frequency questionnaire, as well as from a limited 24-hour recall. Specifically, we hypothesized that a group of women who believed that the study intent was to measure the intake of (healthy) fruits and vegetables might report higher intakes of fruits and vegetables than subjects in a control group, who would believe that the study had a more general purpose. We also hypothesized that responses to the food frequency questions might be more biased than responses to the 24-hour recall.

## Methods

### Design

This study was carried out as a blinded randomized controlled trial. All subjects were recruited by telephone before random assignment to study groups. At the time of recruitment, subjects were asked if they would complete a short phone interview about food intake and health at a future date for a study of eating habits of women living in Colorado. Upon verbal consent to participate, each subject was then told she would soon receive a letter in the mail followed by a telephone interview within ten days. No specific appointments were made regarding the date or time of the telephone interview. Each subject was instructed to read the letter and have it accessible during the interview.

Approximately 2–3 days after recruitment, each subject received a letter in the mail reminding her of the upcoming phone interview and providing information about serving size definitions of foods. Prior to that letter, subjects were randomized 1:1 to the intervention or control group. Letters received by the intervention group were printed on letterhead with colorful graphics of fruits and vegetables and included a small amount of text prompting participants about the health benefits of fruits and vegetables and recommendations to consume 5–9 servings of fruits and vegetables per day ("Colorful fruits and vegetables provide a wide variety of vitamins, minerals, fiber, and phytochemicals your body uses to maintain good health and energy levels, protect against the effects of aging, and reduce the risk of cancer and heart disease. Americans should consume 5–9 servings of fruits and vegetables daily. Eating more fruits and vegetables may be one of the easiest things you can do to improve your health."). Intervention subjects also received a 5-A-Day refrigerator magnet and a 5-A-Day sticker was placed on the outside of the envelope. Letters received by the control group were identical to those received by the intervention group except that they were printed on black-and-white University of Colorado Health Sciences Center letterhead, and they did not include the above text about fruits and vegetables, the 5-a-Day the sticker, or the 5-A-Day magnet.

### Subjects

Women residing in the State of Colorado were randomly selected for recruitment from a commercial database of listed telephone households, specifically targeting females, aged 35–64 (Survey Sampling, International). This commercial database is drawn from telephone directories, school registration lists, magazine subscription lists, voter registration lists, and driver's license lists. Recruitment phone call attempts were made to 663 randomly selected households from this list. Of the phone numbers attempted, 26% were unreachable after seven attempts and 13% of the phone listings were non-working numbers or the intended person did not reside in the household. One percent of the women contacted were ineligible for the study according to their age or did not speak English. Among the 396 women who were contacted and eligible for the study, 206 (52%) agreed to take part. After recruitment, we were unable to contact 34 women (18 controls, 16 intervention subjects) for the dietary assessment. Five women refused at the time of the interview (2 controls, 3 intervention subjects) and 4 were lost to follow up for other various reasons (1 control, 3 intervention subjects). Therefore, 163 women completed the follow-up phone interview (83 controls, 80 intervention subjects).

Phone interviews were typically completed within ten days after the subjects received the letter. The interviews were not scheduled. The phone interviews completed in this study were conducted by trained, professional interviewers, calling at random times, with the interviewer being blinded as to which group each subject was assigned. The phone interview consisted of a standard introduction and preparation. Each subject then responded to an 8-item food frequency questionnaire, a brief 24-hour recall specific to fruits and vegetables, demographic questions, questions regarding health status, and a question about personal beliefs regarding the relationship between fruit and vegetable intake and disease modelled after Trudeau et al [8]. The protocol for this study was reviewed and approved by the Colorado Multiple Institutional Review Board.

### Food frequency assessment

Eight questions from the Behavioral Risk Factor Surveillance System (BRFSS) were used to assess fruit and vegetable intake [9]. In addition, we included the BRFSS questions regarding intake of fluid milk and sweet bread products (doughnuts, cookies, cakes, pastries, and pies), which were not mentioned in the biasing prompt, and are generally considered neutral (milk) or socially undesirable (sweets) to determine whether any bias we might observe would be specific to fruits and vegetables. Subjects responded with the number of times per day, week, month, or year they consumed each food. From these reports, the total number of servings per day of fruits and vegetables was determined.

### 24-hour recall

A brief version of the 2001 California Dietary Practices Survey was used to assess fruit and vegetable intake on the previous day [10]. This recall was a series of eight questions regarding whether the respondent ate each of three meals or a snack the previous day, and (if eaten) whether fruits and/or vegetables were consumed at each of those occasions.

Subjects were also asked questions from the Behavioral Risk Factor Surveillance System interview [11] about factors shown by others to be related to fruit and vegetable intake. These included age, race/ethnicity, education, marital status, smoking, and health status [10-14]. In addition, subjects responded to a question regarding how long it had been since their last doctor's visit for a routine check-up.

### Analysis

We used the two-sample t-test for independent samples to evaluate differences in mean values of continuous variables (e.g., age and number of servings of fruits and vegetables per day) between the randomly-assigned study groups. The chi-square test was used to analyze the categorical variables of interest. Multiple linear regression analysis was used to assess the independence of the association between fruit and vegetable intake and the intervention group, after adjusting for covariates (e.g., education, marital status). Multiple logistic regression was used to assess the independence of associations between intervention group and the categorical outcomes of reported fruits and vegetables servings per day after adjusting for potential confounding factors. SAS, Version 8.0 was used to conduct these analyses [15].

## Results

Control and intervention subject groups were similar with respect to most characteristics (Table 1). The majority of participants in both groups were Non-Hispanic White and married, and perceived their health as very good or good, had received a medical check-up within the last year, and were non-smokers. A slightly higher proportion of women randomly assigned to the intervention group were Non-Hispanic White (96% vs. 88%, *P *= 0.03) and college graduates (64% vs. 44%, *P *= 0.05) than were women randomly assigned to the control group.

**Table 1:** Summary of demographic and health characteristics of intervention and control groups

		Control Group n = 83	Intervention Group n = 80	p-value
Age (years)				0.07
	< 45 years	31 %	26 %	
	45–55 years	51 %	40 %	
	> 55 years	18 %	34 %	
Race/ethnicity				0.03
	Non-Hispanic White	96 %	88 %	
	Other	4 %	12 %	
Education				0.05
	Some HS or HS Grad	24 %	15 %	
	Some College	31 %	21 %	
	College Grad or Higher	44 %	64 %	
Marital Status				0.14
	Married	90 %	82 %	
	Not Married	10 %	18 %	
Smoking Status				0.93
	Smokers	12 %	12 %	
	Non-smokers	88 %	88 %	
Self-Perceived				0.86
Health Status	Excellent	26 %	26 %	
	Very Good/Good	65 %	68 %	
	Fair/Poor	8 %	6 %	
Time Since Last				0.19
Medical Exam	Within Past Year	84 %	76 %	
	More Than One Year Ago	16 %	24 %	

Subjects assigned to the intervention group reported a significantly higher mean intake of total fruits (including juice) (2.0 vs. 1.3 servings per day, *P *< 0.001), total vegetables (3.2 vs. 2.4, *P *< 0.001), and total fruits and vegetables (5.2 vs. 3.7, *P *= < 0.001) than did the control subjects (Table 2). No difference was observed between the intervention and control groups with regard to reports of intake of milk, potatoes, or sweet products (doughnuts, cookies, cake, pastries, or pies) (Table 2). A higher proportion of intervention subjects were categorized as eating 5 or more servings of fruits and vegetables per day by the FFQ (40% vs. 18%, *P *= 0.002 (Table 3). Adjustment by multiple logistic regression for age, education level, race/ethnicity, self-perceived health status, and time since last check up had little effect on this association (Crude OR = 3.0, 95% CI 1.5 to 6.2; adjusted OR = 3.0, 95% CI 1.4 to 6.4) (Table 3). Multiple linear regression analyses also showed no confounding by age, education level, race, self-perceived health status, and time since last medical check-up on servings per day (data not shown).

**Table 2:** Reported mean intake of fruits, vegetables, and other foods collected by food frequency questionnaire

	Mean Servings Per Day (± SD)			
		
	Control Group n = 83	Intervention Group n = 80	Difference	*P*-value
Milk	0.70 (0.72)	0.88 (0.94)	0.18	0.18
Doughnuts, Cookies, Cake, Pastries, or Pies	0.32 (0.5)	0.35 (0.58)	0.03	0.66
100% Fruit or Vegetable Juice	0.43 (0.5)	0.64 (1.04)	0.21	0.10
Fruit	0.87 (0.69)	1.33 (0.95)	0.46	< 0.001
Carrots	0.30 (0.44)	0.38 (0.34)	0.08	0.21
Potatoes	0.24 (0.28)	0.23 (0.22)	0.01	0.97
Green Salad	0.66 (0.46)	0.83 (0.54)	0.17	0.03
Other Vegetables	1.2 (0.84)	1.8 (1.46)	0.6	0.001
Total Fruit and Juice	1.3 (0.89)	2.0 (1.47)	0.7	< 0.001
Total Vegetables	2.4 (1.26)	3.2 (1.86)	0.8	< 0.001
Total Fruits and Vegetables	3.7 (1.66)	5.2 (2.67)	1.5	< 0.001

**Table 3:** Reports of fruits and vegetables intake by food frequency and 24 hour recall methods.

	Control Group (n = 83)	Intervention Group (n = 80)	Crude OR (95% CI)	Adjusted OR^§ ^ (95% CI)
FFQ report of five or more fruit & vegetable servings per day	18 %	40 %	3.0* (1.5 to 6.2)	3.0 (1.4 to 6.4)
				
24 hour recall of fruits or vegetables intake the previous day at:				
breakfast	46 %	64 %	2.0** (1.1 to 3.7)	1.6 (0.85 to 3.1)
lunch	59 %	74 %	1.8** (.96 to 3.5)	1.5 (.77 to 3.1)
dinner	69%	89%	3.6** (1.2 to 7.8)	3.8 (1.6 to 9.4)
snacks	25%	41%	1.8** (1.0 to 3.1)	1.8 (.91 to 3.1)
Sum of three or more times throughout the day	32%	61%	3.4** (1.9 to 6.1)	2.8 (1.6 to 5.2)

*OR estimated via multiple logistic regression as the odds of 5 or more servings per day vs. fewer than 5 for intervention group as compared to control group.

** OR is the odds of having reported eating a fruit or vegetable in the specific meal (yes vs. no), or 3 or more times in the day (vs. under 3 times), compared to the control group, as estimated by logistic regression.

^***§ ***^Adjusted by multiple logistic regression for age, education level, race/ethnicity, self-perceived health status, and time since last medical check-up.

In responding to the 24-hour recall questions, a greater proportion of intervention subjects reported consumption of fruits and/or vegetables the preceding day at breakfast (64% vs. 46%), lunch (74% vs. 59%), dinner (89% vs. 69%), and for snacks (41% vs. 25%) (Table 3). However, only with regard to the dinner meal was the difference statistically significantly higher among the intervention subjects. In total, however, a significantly greater proportion of intervention subjects reported consumption of fruits and/or vegetables on three or more occasions throughout the previous day than did control subjects (61% vs. 32%, adjusted OR = 2.8, 95% CI 1.6 to 5.2).

A higher proportion of subjects in the intervention group considered their diets very high or high in fruits and vegetables than the control group (61% vs. 39%, *P *= 0.05).

## Discussion

In this study, intervention subjects exposed to brief messages about the benefits of fruits and vegetables and 5-A-Day guidelines for consumption in the day preceding dietary assessment reported substantially higher intakes of fruits and vegetables by both the FFQ and the 24-hour recall methods. Adjustment for factors known to be associated with fruit and vegetable intake had little effect upon the size of this difference. This difference in behavior could reflect an actual (although most likely temporary) change in behavior or an intention to change. However, the minimal nature of the intervention and the large size of the difference make this explanation unlikely. This study therefore suggests that social approval bias might well be a substantial problem in the interpretation of nutritional intervention effects that are dependent on education and awareness to affect behavior change. The magnitude of this bias is similar to the intervention effects reported in many studies evaluating changes in fruit and vegetable intake (ranging from 0.93 to 1.25 servings per day) [16-18]. Thus, a major challenge facing nutritional intervention researchers is assessing true behavioral change based on self-reports from reporting bias.

This study employed brief dietary assessment methods that are commonly used in nutritional epidemiologic studies, including intervention trials [16-19]. The estimates of mean fruit and vegetable intake per day among control subjects from the food frequency portion of the interview was 3.71 servings, which is consistent with baseline or control mean intake amounts identified in other studies as well as the National Cancer Institute 5-A-Day program evaluation [19-21]. In addition, we observed an association between fruit and vegetable intake and self-perceived health status that is consistent with findings of previous research [10].

We had hypothesized that the 24-hour recall might be less susceptible to the effects of social approval bias because answers are reported according to memory of the previous day's intake instead of being derived from more generic memory, as in the food frequency questionnaire [7]. However, we observed substantial biases in both types of measures. More intervention subjects reported consumption of fruits and vegetables than did controls for each meal on the prior day, and substantially more therefore reported eating fruits and vegetables on 3 or more occasions on the previous day (61% vs. 32%, p = 0.002). These findings suggest that social approval bias may affect short-term dietary recall methods as well as more general longer-term recall processes used for food frequency reports.

Our findings are consistent with previously established theories regarding the effects of social desirability and social approval bias upon self-reports of usual food intake. Prompting subjects in the intervention group about the benefits of fruits and vegetables likely altered their memory of food intake to be consistent with what is considered "good for health". Responses about other food items included in the food frequency questionnaire, such as milk, potatoes, and sweet bread products, that may be considered neutral or socially undesirable, were unaffected by this apparent bias.

Results of this study were obtained from a small sample of the Colorado female population. We were therefore unable to assess the impact of reporting bias among men, or among subgroups defined by age, race/ethnicity, or socioeconomic status. Future work should examine effects in such subgroups, and should also assess the relationship between the dose and timing of the biasing prompts, and the resulting bias. More detailed assessments of full 24 hour diet recalls (rather than only the fruit and vegetable specific limited recall used in this study) should also be examined in future studies. Future work should also examine the bias in reports of diet over time between intervention and control subjects, where change is measured as the difference in the intervention-control differences over time. Any difference in fruits and vegetables intake between baseline and follow up should be interpreted with caution as it could be due to either bias from participating in the intervention, or to actual change.

Another explanation of the difference in reporting fruits and vegetables intake reported in this study between the study groups could be that the diets between these two groups were really different, and not just on the day of the interview. However, the randomized blinded nature of this study makes this explanation unlikely. The difference in the reported fruits and vegetables intake can also be attributed to the intervention resulting in a temporary behavior change in the intervention group. We believe it is unlikely that such a small "intervention" could account for such a large behavioural change.

Alternatives to self-reported measures of dietary intake in the evaluation of dietary interventions deserve close attention and careful thought in the design of dietary intervention studies. Methods of data collection that are more objective in nature will reduce bias and provide more reliable estimates of intervention effects [22]. Alternatives for consideration may include use of nutritional biomarkers such as carotenoids to assess changes in nutritional intake throughout the intervention process, as used in the Polyp Prevention Trial [23] and in an assessment of fruit and vegetable intake in Norway [24]. Although use of nutritional biomarkers may exhibit some limitations due to cost and compliance, this more objective method of dietary assessment eliminates the concerns about bias associated with self-reported dietary intake [25,26].

In some settings, where intervention targets are specific to confined groups of people, direct observation of dietary intake before and after an intervention may be feasible as an objective method of dietary assessment. For example, direct observation of children eating in school settings, or workers in worksite cafeterias can provide an objective dietary measure for interventions expected to affect food choices. For community-wide interventions, evaluation of food purchases at markets or purchasing trends at dining establishments are objective measures for interventions expected to change purchasing patterns.

In large dietary intervention trials, comprehensive solutions to eliminate the bias in self-reported dietary intake may prove costly and unrealistic for all subjects. However, subgroups can be assessed with biomarkers or other independent assessments to at least estimate the size of the reporting bias that might be expected to have been induced by the intervention. Bias can also be controlled to some degree by evaluating different intensities of an intervention, in which everyone receives at least a minimal prompt for change, rather than evaluating differences between study groups exposed to an intense intervention as compared to those not exposed to any intervention.

## Conclusion

Our results show that self-reports of fruit and vegetable intake by means of both food frequency questionnaires and 24-hour recall are susceptible to substantial social approval bias. Attention to this bias in the context of dietary reports from subjects in nutritional intervention studies is an important consideration in study design, analysis, and interpretation. Continued efforts to improve methods to objectively evaluate nutritional interventions are needed.

## Competing interests

The authors declare that they have no competing interests.

## Authors' contributions

TMM designed and coordinated the study, recruited subjects, collected data, analyzed data, wrote the manuscript, and helped edit the manuscript, MFA helped design the study, provided significant professional consultation, recruited subjects, collected data, and helped edit the manuscript, LAC helped design the study, edit the manuscript, and provided consultation, ACM helped design the study, helped with the study coordination and editing the manuscript, and provided study consultation, TEB helped design the study, provided significant professional advice, and helped write and edit the manuscript.
